# Functionally different α-synuclein inclusions yield insight into Parkinson’s disease pathology

**DOI:** 10.1038/srep23116

**Published:** 2016-03-17

**Authors:** Christian C. Raiss, Theresa S. Braun, Irene B. M. Konings, Heinrich Grabmayr, Gerco C. Hassink, Arshdeep Sidhu, Joost le Feber, Andreas R. Bausch, Casper Jansen, Vinod Subramaniam, Mireille M. A. E. Claessens

**Affiliations:** 1 Nanobiophysics Group, MESA+ Institute for Nanotechnology & MIRA Institute for Biomedical Technology and Technical Medicine, Faculty of Science and Technology, University of Twente, Enschede, The Netherlands; 2 Biomedical Signal and Systems, and Neurophysiology, MIRA Institute for Biomedical Technology and Technical Medicine, University of Twente, Enschede, The Netherlands; 3 Laboratorium Pathologie Oost-Nederland, Postbus 516, 7550 AM Hengelo, The Netherlands; 4 Lehrstuhl für Zellbiophysik E27, Technische Universität München, 85748 Garching, Germany; 5 FOM Institute AMOLF, Science Park 104, 1098 XG Amsterdam, The Netherlands

## Abstract

The formation of α-synuclein (α-S) amyloid aggregates, called Lewy bodies (LBs), is a hallmark of Parkinson’s disease (PD). The function of LBs in the disease process is however still unclear; they have been associated with both neuroprotection and toxicity. To obtain insight into this contradiction, we induced the formation of α-S inclusions, using three different induction methods in SH-SY5Y cells and rat-derived primary neuronal cells. Using confocal and STED microscopy we observed induction-dependent differences in α-S inclusion morphology, location and function. The aggregation of α-S in functionally different compartments correlates with the toxicity of the induction method measured in viability assays. The most cytotoxic treatment largely correlates with the formation of proteasome-associated, juxta-nuclear inclusions. With less toxic methods cytosolic deposits that are not associated with the proteasome are more prevalent. The distribution of α-S over at least two different types of inclusions is not limited to cell models, but is also observed in primary neuronal cells and in human mesencephalon. The existence of functionally different LBs, *in vivo* and *in vitro*, gives important insights in the impact of Lewy Body formation on neuronal functioning and may thereby provide a platform for discovering therapeutics.

The aggregation of soluble proteins into insoluble, β-sheet-rich amyloid fibrils is characteristic for many neurodegenerative diseases. Intraneuronal aggregates of α-synuclein (α-S) are for example found in Parkinson’s disease (PD), Lewy body dementia and multiple system atrophy^1^,^2^,^3^. Whereas extracellular β-amyloid deposits and intracellular accumulations of phosphorylated tau protein occur in Alzheimer’s disease^4^,^5^. In Huntington’s disease, polyglutamine-expanded huntingtin (htt) protein accumulates within intranuclear inclusion bodies or neurites^6^ and in amyotrophic lateral sclerosis, motor neurons develop protein-rich inclusions containing superoxide dismutase 1, TAR DNA-binding protein 43 or the RNA-binding protein fused-in-sarcoma in their cell bodies and axons^7^,^8^,^9^,^10^.

In PD, α-S amyloid inclusions such as Lewy neurites (LN) and Lewy bodies (LB) can be found in neurons and glia cells^2^,^3^,^11^. The topographical progression of neuronal death, and the development of α-S immunoreactive Lewy body related structures^12^,^13^, here abbreviated to Lewy body like inclusions (LBLI), throughout the brain is used to stage PD pathology^14^. The pathologically determined stages are in many cases related to clinical features observed in patients^15^. Nevertheless, the role of LBLI during the progression of PD is unclear. LBLI may be indicative of cellular dysfunction and death^16^,^17^ but have also been described as harmless, inert or neuroprotective protein aggregates^18^. PD symptoms have been shown to directly correlate with the density of neurons in the substantia nigra pars compacta^19^, but no correlation could be established between the number of LBLI and the severity of disease symptoms^16^. Assuming LBLI are indeed inert, one would expect the affected cells to have a normal life span. With the loss of other cells in the tissue^19^, the proportion of cells with LBLI should therefore increase. However, recent studies show that in the brain of PD patients the proportion of cells with such inclusions is constant^17^. It is therefore likely that LBLI do play a role in neuronal death. Yet surprisingly, LBLI are also observed in individuals not showing PD symptoms^20^,^21^. Thus, the role of these inclusions in the progression of PD is disputed, and the question remains if LBLI are toxic or the effect of a neuroprotective response to possibly toxic protein misfolding.

Recent studies propose that the process of protein aggregation is regulated and misfolded proteins are sequestered to inclusion sites with distinct functions^22^. In protein aggregation studies in cell model systems, two intracellular compartments for the sequestration of misfolded cytosolic proteins were identified: the JUxta Nuclear Quality control compartment (JUNQ) and the insoluble protein deposit (IPOD)^23^. The JUNQ inclusion site is located adjacent to the nucleus. It encloses soluble, ubiquitinated, misfolded proteins and chaperones mediating refolding or degradation. The JUNQ compartment is used to temporarily store misfolded proteins. Amyloidogenic proteins that cannot be degraded or refolded are sequestered to the IPOD. The degradation or refolding of proteins in the JUNQ can be hampered when it becomes saturated with insoluble aggregates^23^. It has been demonstrated that when this happens JUNQ inclusions turn neurotoxic^24^.

Here, we induced α-S aggregation and the formation of α-S inclusions in a cell model system to comprehend the possible origin of toxicity or protection of α-S amyloid inclusions. We used three different induction protocols which resulted in significantly different cytotoxicity. We demonstrate a dependence of α-S inclusion morphology and location on induction method and show, to the best of our knowledge for the first time, that α-S can sequester to- and aggregate in- functionally different compartments *in vivo* and *in vitro*. We hypothesize that accumulation of α-S in these different cellular compartments determines cell fate and sheds light on the disputed role of LBLIs in PD.

## Results

### *In vitro* expressed α-S-GFP can be incorporated in amyloid fibrils

To enable visualization of α-S distribution and aggregation in SH-SY5Y cells, α-S was tagged with green fluorescent protein (α-S-GFP). To exclude the possibility that the attached GFP-tag hinders α-S fibrillization, the fibril-forming properties of α-S-GFP were assessed *in vitro*. For this purpose, recombinant α-S-GFP was expressed in *E. coli* (Fig. S1), isolated and tested for fibrillization. At physiological salt concentrations, purified-untagged, recombinantly expressed α-S assembles into amyloid fibrils^25^,^26^,^27^. Fibrillization could not be observed for pure α-S-GFP or 1:1 ratios of tagged and untagged α-S. After 3 days of incubation in a 1:10 ratio however, α-S-GFP fibrils were clearly detectable by fluorescence microscopy. A control experiment in which untagged α-S was aggregated and labelled with Thioflavin T indicates that compared to fibrils of untagged α-S the α-S-GFP containing fibrils may be shorter (Fig. S2). Under the experimental conditions used, long time incubation of GFP alone did not result in fibril formation (data not shown).

### α-S-GFP expression in cells

To effectively visualize the distribution of α-S monomers and aggregates inside SH-SY5Y cells, an α-S-GFP fusion construct was stably transfected. Both untagged-endogenous and GFP-tagged α-S were detected by Western blot analysis of total lysate from differentiated α-S-GFP SH-SY5Y cells (Fig. S3A). Total lysate from control SH-SY5Y cells without the fusion construct only contained untagged α-S. In the stably transfected cells, endogenous-untagged α-S was present in amounts close to the detection limit and could only be observed by increasing the contrast of the blots significantly. α-S-GFP from the transfected construct was clearly visible (see Supplementary Fig. S3A). No free GFP could be detected in the cell lysates (Fig. S4). Fluorescence microscopy of α-S-GFP SH-SY5Y cells showed that all cells express α-S-GFP. However, different α-S-GFP fluorescence intensities were observed in the cell population (see Supplementary Fig. S3B). The differences in intensities were attributed to different expression levels of α-S-GFP and therefore probably different ratios of untagged α-S to α-S-GFP. We observed a diffuse distribution of α-S-GFP throughout the cells. Immunolabelling of α-S showed both a cytosolic and an intranuclear expression pattern (see Supplementary Fig. S3C). Further, we were interested in the effect of α-S-GFP on cell morphology. Visualization of the actin network with fluorescently tagged phalloidin in control cells and cells expressing α-S-GFP did however not reveal notable differences (data not shown).

### α-S seeds nucleate α-S aggregation *in vitro*

The expression of α-S or α-S-GFP in SH-SY5Y cells did not by itself result in the formation of α-S inclusion bodies on the time scales studied. One way to initiate α-S monomer aggregation and shorten the aggregation lag time, is adding seeds to a pool of monomeric α-S protein^28^. In cells, α-S-fibril seeds may recruit cytoplasmic soluble α-S proteins and convert them to the insoluble β-sheet rich fibrils. Hence, we produced α-S seeds by sonication of pre-aggregated α-S fibrils. The resulting fibril fragments were characterized using tapping mode atomic force microscopy (AFM). The obtained AFM images show a population of fibrils with lengths up to 500 nm (see Supplementary Fig. S5A). To evaluate the potency of the created α-S seeds to initiate α-S aggregation, we added them to a pool of monomeric α-S and followed amyloid formation in a Thioflavin T (ThioT) fluorescence assay. The control containing only α-S monomers did not show aggregation in the first 24 hours (see Supplementary Fig. S5B). The addition of fibril seeds circumvented the slow formation of aggregation prone nuclei. Amyloid growth started immediately after seed addition.

**Figure 1:**
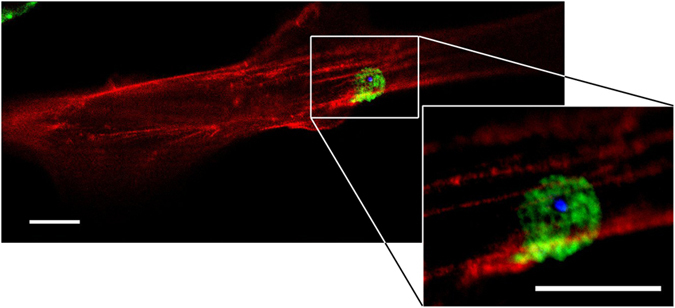
α-S-seeds nucleate the formation of α-S inclusions in SH-SY5Y cells. Differentiated SH-SY5Y cells were exposed to 500 nM fluorescently labelled α-S seeds (α-S-seeds^ThioT^: blue) and immunostained for α-S (α-S^alexa594^: green). After this treatment, the α-S was, in some cells, no longer homogenously distributed over the cell volume but concentrated in micrometer sized inclusions. A magnification of the inclusion shows that α-S-seeds^ThioT^ were taken up by the cell and form the core of an inclusion. Scale bar: 3 μm, STED microscopy.

To be able to initiate α-S aggregation inside cells, α-S seeds must be internalized and come in contact with the endogenous α-S pool. Previous studies have shown that amyloid fibrils can be internalized by cells after addition to culture medium^29^,^30^,^31^,^32^. Accordingly, we administered α-S seeds to the cell medium. Using confocal microscopy, we mainly observed attachment of α-S fibril clusters to the outer cell membrane. A fraction of the fibril clusters was internalized within the first 12 hours (see Supplementary Fig. S6). We further observed that intracellular inclusion formation could be nucleated by internalized α-S fibrils. In wild type SH-SY5Y cells, we observed α-S inclusions formed around the internalized fluorescently-labelled α-S seeds. The seeds apparently served as a nucleation site to which endogenous α-S was recruited (Fig. 1).

### α-S inclusion characteristics and morphology

**Figure 2:**
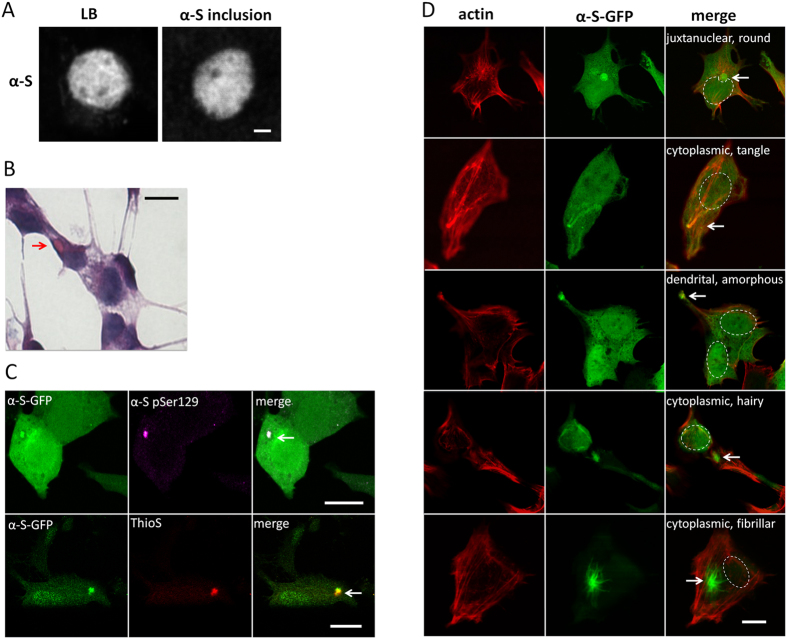
Induced α-S inclusions show key features of LBLIs. (**A**) LBLIs in human mesencephalon derived from a PD patient and α-S inclusions in α-S-GFP SH-SY5Ycells both contain α-S, are comparable in size, α-S density and morphology; grey: α-S^alexa594^, STED microscopy; scale bar: 0.5 μm. (**B**) Haematoxylin/Eosin staining reveals eosinophilic oval α-S inclusions in α-S-GFP SH-SY5Y cells; an inclusion is indicated by the red arrow, bright field microscopy, scale bar: 10 μm. (**C**) α-S in inclusions is phosphorylated and binds ThioS; green: α-S-GFP, purple: α-S pSer129^alexa555^, red: ThioS, α-S inclusions indicated by white arrow, scale bar: 10 μm. (**D**) Different inclusion morphologies (hairy, amorph, tangle, fibrillar, round) were observed in different cellular locations; α-S-GFP (green), actin^alexa647^ (red); nuclei are indicated by the dashed lines; confocal images, scale bar: 10 μm.

Besides the addition of 500 nM α-S seeds we used two other approaches to induce the formation of α-S inclusions in SH-SY5Y cells: i) addition of 100 μM α-S monomers, and ii) exposure to 5 nM rotenone^33^. We compared the induction approaches at time points where, upon visual inspection, comparable fractions of the cells contained α-S inclusions. For α-S inclusions induction using α-S monomers and α-S seeds this was usually after 5 days while cultures needed to be exposed to rotenone for 4 weeks to obtain a comparable fraction of α-S inclusions. The thus obtained inclusionts were comparable in size and morphology to LBLI observed *in vivo* (Fig. 2A) and displayed key features of LBs and LNs, such as phosphorylation of α-S and eosinophilia (Fig. 2B,C). Additionally, we observed Thioflavin S (ThioS) binding to the α-S inclusions, indicating that they consist of cross-beta sheet fibrils (Fig. 2C) The morphology of the α-S inclusions obtained varied from LB-like spherical inclusions to spindle or thread like aggregates resembling LNs. The inclusions differed in α-S density and distribution and were observed in both the cell body and cell extensions (Fig. 2D). The morphology of α-S inclusions found in differentiated α-S-GFP SH-SY5Y cells was comparable to those present in differentiated wild type SH-SY5Y cells and rat primary neuronal cells (data not shown). Similar α-S inclusion morphologies were observed in *in vivo* in human brain (see Supplementary Fig. S7). Neuropathological α-S inclusions are often difficult to characterize into e.g. classical Lewy bodies, cortical Lewy bodies and pale bodies. The outcome of such a characterization depends on the treatment and antibodies used and additionally there is a huge inter-observer variability^12^,^13^. To circumvent ambiguities we therefore classified all the α-S inclusions and quantified differences in morphology and location using physical parameters (see Materials and Methods).

**Figure 3:**
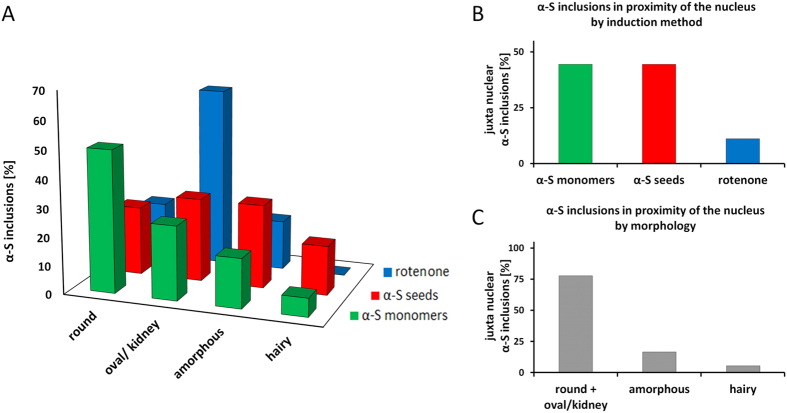
The induction method determines α-S inclusion morphology and proximity to the nucleus. (**A**) α-S inclusions were induced in SH-SY5Y cells and α-S-GFP SH-SY5Ycells with rotenone (blue, N = 58), α-S monomers (green, N = 46), α-S seeds (red, N = 46) and categorized based on their morphology (round, oval, amorphous, hairy). For each condition 20 inclusions in GFP-SH-SY5Y cells were taken into account, the remainder of the data was obtained in SH-SY5Y cells. α-S inclusions induced by α-S monomers are predominantly round (50%). When treated with α-S seeds, all inclusion morphologies appear in a comparable amount. Induction with rotenone mainly results in oval inclusions (63%). (**B**) Approximately 44% of the α-S inclusions induced with α-S monomers (green) and α-S seeds (red) are juxtanuclear (N = 36). After treatment with rotenone (blue) only 11% of the inclusions are found close to the nucleus. (**C**) α-S inclusion morphology is a good predictor for inclusion location in the cell. Most juxtanuclear inclusions are either round or oval/kidney-like (78%). The probability for amorphous (17%) and hairy (6%) α-S inclusions to be close to the nucleus is low.

Interestingly, the obtained α-S inclusion morphology and location in the cells differed between the three induction methods. Generally, α-S inclusions induced by the addition of α-S monomers were spherical and often localized near the cell nucleus. Treatment with rotenone mainly resulted in oval/kidney-like morphologies with well-defined borders. After exposure to fibrillar α-S seeds, a considerable fraction of cells contained cytoplasmic thread-like, radiating fibrillar or less dense and diffuse inclusions. Such hairy inclusions were largely absent after the other induction approaches. Similar results were obtained for wild type SH-SY5Y cells, α-S-GFP SH-SY5Y cells (Fig. 3A) and primary neuronal cells (see Supplementary Fig. S8).

To quantify the different shapes, we classified the obtained α-S inclusions based on their morphology and their proximity to the nucleus. Figure 3A summarizes the distribution of the observed inclusion morphologies in wild type SH-SY5Y and α-S-GFP SH-SY5Y cells for all three induction treatments. The morphologies were classified in round, oval/kidney-like, amorphous or hairy (fibrillar threads). Most inclusions induced by α-S monomers were round (50%), with smaller fractions of oval (26%) and amorphous (17%) LBLIs. We could only find a minor fraction of hairy inclusions (7%). After treatment with α-S seeds comparable amounts of all α-S inclusion morphologies were found: round 24%, oval 29%, amorphous 29%, hairy 17%. Induction of α-S inclusion formation with rotenone mainly resulted in oval inclusions (63%). The remainder of the inclusions found after rotenone treatment were round (20%) or amorphous (17%); hardly any hairy inclusions were present.

The different induction methods not only influenced α-S inclusions morphology, but also the cellular location of the inclusions. We therefore also classified inclusions by their proximity to the cell’s nucleus. In contrast to the induction with rotenone where only 11% of all inclusions were found in proximity of the nucleus, 44% of all inclusions formed after treatment with α-S seeds or α-S monomers were found close to the nucleus (Fig. 3B). Further, we determined which α-S inclusion morphology was most likely found close to the nucleus. 78% of the α-S deposits located in proximity of the cell’s nucleus were round or oval/kidney-like. In contrast, 83% of amorphous inclusions and 94% of hairy α-S deposits were found further away from the nucleus, in the cytoplasm (Fig. 3C). We speculate that different factors like the availability of endogenous α-S, crowding, or colocalization with other proteins and cellular structures may define the α-S inclusion morphologies. Especially after induction with α-S seeds a relatively large fraction of inclusions with a hairy morphology was observed. These fibrillar α-S accumulations span the cytoplasm and might have developed along cytoskeletal networks.

### α-S sequesters in functionally different α-S inclusions

**Figure 4:**
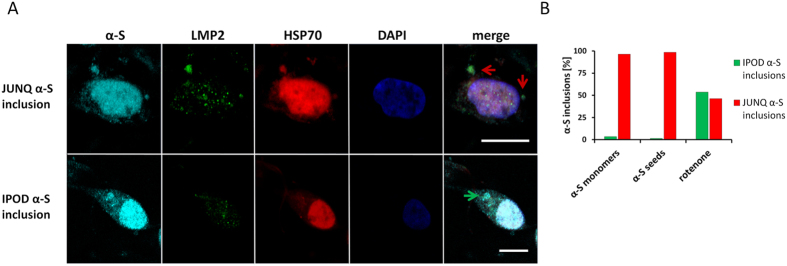
α-S sequesters in IPOD and JUNQ- inclusions in SH-SY5Y cells. The ratio of IPOD to JUNQ is dependent on the induction method. (**A**) α-S inclusions are induced with 5 nM rotenone for 4 weeks in SH-SY5Y cells. Immunostaining was performed for α-S^alexa555^ (cyan), lmp2^alexa488^ (green), hsp70^alexa633^ (red) and the nucleus was visualized using DAPI (blue). Based on protein colocalization, two different α-S inclusion types can be observed. hsp70 and lmp2 colocalize with α-S in JUNQ (top row, red arrows), in other α-S inclusions, no colocalization with these proteins can be observed and they are therefore categorized as IPOD (bottom row, green arrow); scale bar: 10 μm. (**B**) After treatment with 100 μM α-S monomers (N = 57) or 500 nM α-S seeds (N = 68) for 24 h, many JUNQs (96,5% and 98,5%) developed in cells. In contrast, after 5 nM rotenone treatment for 4 weeks, 54% of IPODs were observed (N = 54).

Intracellular protein aggregation is considered a well-managed process^23^,^34^. In this context, two intracellular inclusion sites were described: the “juxta-nuclear quality control” compartment (JUNQ) is an intracellular region in which soluble misfolded proteins concentrate to be re-folded by chaperones or to be prepared for degradation by the proteasome. Insoluble and non-degradable amyloids are sequestered to the cytoplasmic “insoluble protein deposit” (IPOD). These inclusion sites can be distinguished by their colocalization with distinct proteins. The chaperone heat shock proteins 70 (hsp70) and the proteasome subunit low molecular mass protein 2 (lmp2) are both typically active in the JUNQ and absent in the IPOD. Accordingly, we assigned α-S inclusions to functionally different compartments, based on their colocalization with hsp70 and lmp2 in wild type SH-SY5Y cells (Fig. 4A), α-S-GFP SH-SY5Y cells (data not shown) and rat primary neuronal cells (see Supplementary Fig. S9). For all three α-S inclusion induction approaches both JUNQ and IPOD-deposits were identified, but distribution of α-S inclusions over these compartments differed. A significantly higher amount of JUNQ-deposits was obtained after the α-S monomer and α-S seed treatments, compared to the treatment with 5 nM rotenone. Only 4% of α-S inclusions found after treatment with α-S monomers and 2% after treatment with α-S seeds appear to be IPOD-inclusion. In contrast, after treatment with rotenone, 54% of total inclusions studied showed characteristics of IPOD inclusions (Fig. 4B). Based on the juxtanuclear location of JUNQ- inclusions, we conclude that JUNQ- inclusion typically have a round or oval/kidney-like morphology (Fig. 3C). In the absence of conclusive immunostaining, location and morphology seem good parameters to assign α-S inclusion to IPOD and JUNQ.

### Functionally different α-S inclusions might affect cellular survival

**Figure 5:**
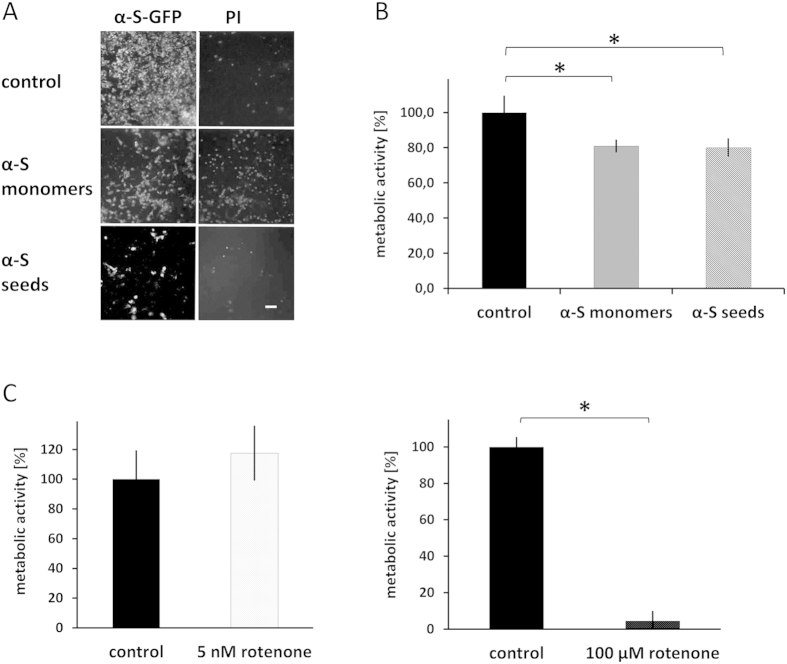
α-S inclusion induction methods differ in cytotoxicity. The viability of α-S-GFP SH-SY5Y cells treated according to the 3 α-S inclusion induction protocols was assessed in viability and metabolic activity assays. (**A**) Exposure to 100 μM α-S monomers or 1 μM α-S seeds lead to decreased cell viability and detachment of α-S-GFP SH-SY5Y cells 72 hours after administration. (**B**) Administration of 100 μM α-S monomers and 500 nM α-S seeds resulted in a significant decrease in metabolic activity after 36 hours. (**C**) A low rotenone concentration of 5 nM in the medium did not have an effect on cell viability even after 4 weeks. In contrast, α-S-GFP SH-SY5Y cells treated with 100 μM rotenone for 36 hours show a significant decrease in metabolic activity; paired student’s t-test: *signifies a p-value < 0.05.

Long-term exposure to 5 nM rotenone leads to inclusion body formation in dopaminergic neurons of animal and human origin^33^. At higher concentrations, rotenone is very toxic (Fig. 5C, right panel). It inhibits the mitochondrial electron transport chain^35^, decreases proteasome activity and increases intracellular reactive oxygen species levels^36^. These elevated levels of reactive oxygen species were found to induce cellular proteins to form insoluble aggregates^37^. α-S aggregation itself, e.g. induced by a high intracellular α-S concentration, has also been related to decreased viability and increased cytotoxicity^32^,^38^,^39^. We therefore compared the viability of cells, treated according to the different α-S inclusion induction protocols, and assessed the metabolic activity in MTT assays.

**Figure 6:**
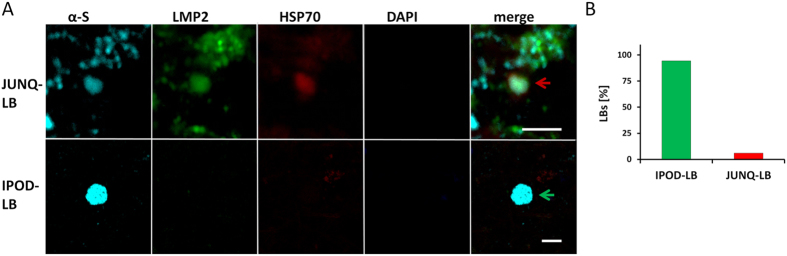
α-S sequesters in different LBLIs. Sample of mesencephalon of a patient suffering from clinical dementia (LB disease). (**A**) Immunostaining was performed for α-S^alexa555^ (cyan), lmp2^alexa488^ (green), hsp70A^alexa633^ (red) and counterstaining was performed with DAPI. Judging LBLI colocalization with lmp2 and hsp70, both IPOD and JUNQ-LBLI can be observed. Whereas IPODs (lower row, indicated by green arrow) can become comparatively large, JUNQs (upper row, indicated by white arrow) are usually smaller and less dense; scale bar: 10 μm. (**B**) In the patient material many more IPODs (94%) than JUNQs were observed (N = 76).

In the presence of 100 μM α-S monomers or 1 μM α-S seeds the uptake of PI, visualized after 72 hours, indicates a decrease in viability. Note that almost all cells exposed to 1 μM α-S seeds detached from the surface, leaving hardly any adherent cells (Fig. 5A). Reducing the concentration to 500 nM α-S seeds kept considerably more of the α-S seed-treated cells attached (data not shown). Already after 24 hours, treatment with 100 μM α-S monomers or 500 nM α-S seeds resulted in a significant reduction in the metabolic activity (Fig. 5B). Treatment with high rotenone concentrations caused massive cell detachment (data not shown) and let to a 93% decrease in metabolic activity. In contrast, such a significant decrease in metabolic activity could not be detected upon treatment with the low rotenone concentrations. Even after 4 weeks in culture with 5 nM rotenone, we did not observe significant differences in metabolic activity compared to the control group (Fig. 5C). Treatment with α-S monomers, α-S seeds and rotenone leads to the formation of functionally different α-S inclusion. Accumulation of aggregated or aggregation prone proteins in JUNQ inclusions has been reported to be cytotoxic^24^. The observed differences in cytotoxicity of the α-S inclusion induction protocols might be directly related to the amount of JUNQ- and IPOD-inclusions obtained with these different methods, but stress factors may also play a role. Speculating that the amount and ratio of JUNQ- to IPOD–inclusions might have a significant effect on cellular survival *in vitro*^24^,^34^, we extended our approach to *in vivo* samples. Using the protocol for distinguishing JUNQ- from IPOD-inclusions *in vitro*, we tested whether similar differences in α-S inclusions can be observed *in vivo*. In diseased human brain tissue, many LBLI in the mesencephalon could be observed after immunolabelling for α-S (data not shown). Additional immunolabelling for hsp70 and lmp2 showed, like for the *in vitro* cell model, two distinct types of α-S inclusions in this patient (Fig. 6A). The majority of the inclusions had IPOD characteristics (96%) whereas only a minor fraction could be characterized as JUNQ- inclusions (4%) (Fig. 6B).

## Discussion

Amyloid deposits represent a hallmark of many neurodegenerative diseases^2^,^3^,^4^,^5^,^6^,^7^,^8^,^9^,^10^,^40^,^41^,^42^. Toxicity in these diseases has however often been attributed to oligomeric protein aggregates^43^,^44^,^45^,^46^. In that respect, it remains controversial whether the amyloid deposits themselves are toxic^6^,^16^,^17^,^18^,^47^,^48^,^49^,^50^,^51^,^52^,^53^,^54^. In some diseases, inclusion body formation seems neuroprotective. For example, studies on Huntington’s disease indicate that the formation of htt inclusion bodies reduced the soluble htt protein and enhanced neuronal survival^54^. On the other hand, in PD, inclusions induced in neurons resulted in propagation of pathological aggregates, selective decrease in synaptic proteins, progressive impairment of neuronal excitability and connectivity and eventually cell death^55^.

Whether inclusion body formation is toxic may depend on several factors. Misfolded proteins have been reported to sequester in different cell compartments^23^,^34^. Protein accumulation and aggregation in these compartments has been related to neurotoxicity^24^. Additionally, heterogeneity in inclusion body morphology, protein composition, density and location in the cell has been reported^23^,^33^,^56^,^57^,^58^. Here we show that the three tested induction methods result in α-S inclusions which differ in morphology, cellular location and colocalization with marker proteins. These marker proteins indicate that, depending on the induction method, the α-S inclusions can appear in different types of protein quality control (PQC) compartments. The toxicity of the induction methods seems to correlate with the distribution of α-S inclusions over these different compartments and we therefore hypothesize that the sequestration of α-S in specific compartments determines cell fate and disease pathology. However, in the experiments reported here we focused on three different methods with comparable amount of inclusion formation. The relation between the concentrations of the compounds used to induce inclusion formation, the types of α-S inclusions formed, and toxicity needs to be further investigated.

The treatment with α-S seeds and α-S monomers significantly reduced cell viability and metabolic activity (Fig. 5A,B). The mechanism by which α-S seeds affect cell fate is not fully understood. However, to serve as nucleation points for α-S aggregation, α-S fibrils must be internalized and gain access to the cytoplasmic α-S monomer pool. Short fibril fragments can enter the cytoplasm, as inclusions consisting of endogenous α-S are shown to originate from externally supplied α-S-fibril fragments (Fig. 1). This implies that α-S amyloid fibril fragments can propagate LB formation and may hence play an essential role in LB formation *in vivo*. In literature, this potential infectious mechanism has been discussed. α-S aggregation has been shown to propagate from mouse brain to grafted dopaminergic neurons and to seed aggregation in human cell cultures, indicating exchange of amyloid material or take up after release of amyloid deposits from dying or dead cells^59^,^60^.

Compared to the α-S inclusion induction method based on the addition of α-S fibril fragments, the concentration of α-S monomers used to induce intracellular protein aggregation was high. This relatively high α-S concentration was used to ensure monomer take up, to distort the well-regulated intracellular α-S concentrations and allow a fast formation of α-S inclusions. Although the concentrations used in the experiments are 2–4 times higher than the reported physiological α-S concentrations, elevated concentrations of α-S are observed in PD. It has been reported that in cases of familial PD with α-S gene locus triplication, the α-S monomer concentration in the brain was significantly higher than normal which can lead to protein aggregation^61^. Further, α-S was shown to be secreted to the extracellular space. There, it can activate and liberate microglia from the surrounding extracellular matrix, which is a hallmark for neuroinflammation^62^. Extracellular α-S can also be endocytosed by astrocytes, which express pro-inflammatory cytokines^62^. These responses could lead to increased stress levels in cultures and may lead to toxicity. At the monomer concentrations used, α-S was observed to aggregate in cell medium after 24–48 hours (see Supplementary Fig. S10). Hence, we cannot exclude the possibility that in the treatment with α-S monomers the induction of α-S inclusion formation, or the observed effect on viability and metabolic activity, results from α-S fibrils or oligomeric intermediates. However, toxicity depends on the particle concentration, not on the equivalent monomer concentration^63^. The particle concentration of the long fibrils in the medium is low compared to that of sonicated fibrils. Moreover, a comparable decrease of metabolic activity is observed when cells are treated with α-S-monomers at concentrations below the critical aggregation concentration^64^, indicating the existence of other, monomer dependent pathways. Of the three approaches used to induce the formation of α-S inclusions, the treatment with a low concentration of rotenone was least toxic. This treatment hardly affected the metabolic activity of the cells over weeks (Fig. 5C). Yet, a comparable number of cells containing α-S inclusions was found, indicating functional differences between α-S inclusions resulting from α-S monomer or α-S seed treatments compared to the low dose rotenone treatment.

Biochemically different inclusion bodies related to protein misfolding have been identified and described in yeast^23^,^34^,^65^ and mammalian cells^24^,^34^,^66^,^67^. To maintain protein homeostasis, misfolded or aggregated proteins need to be refolded, degraded or stored safely to avoid jeopardizing cellular survival. Misfolded proteins have been observed to localize to distinct PQC compartments depending on their solubility. When the proteasome system is saturated by increased protein load, misfolded soluble proteins are temporarily stored in the JUNQ compartment, while insoluble amyloid aggregates are sequestered in IPODs. In protein aggregation diseases, this localization to different PQC compartments has been related to neurotoxicity or neuroprotection. In these diseases, sequestration of misfolded or aggregated proteins in the JUNQ compartment can become toxic. The accumulation of many aggregation-prone proteins may clog the JUNQ compartment, and block the path to proteasomal degradation. Further, JUNQs can sequester chaperones, like hsp70, involved in protein refolding, leading to impairment of cellular protein homeostasis. Misfolded or aggregated proteins may accumulate in the cytoplasm and become toxic^23^,^24^,^68^.

In contrast to results reported for other proteins^23^, α-S was observed to accumulate in both IPOD and JUNQ compartments. The ratio of the distribution of α-S between these compartments depended on the induction method (Fig. 4B). In agreement with the observed cytotoxicity, many JUNQ-inclusions and hardly any IPODs were observed shortly after treatment with α-S monomers or α-S fibrils. IPOD-inclusions were much more prevalent after the less toxic rotenone treatment. Induction of α-S inclusion formation with low concentrations of rotenone resulted in approximately equal amounts of JUNQ- and IPOD-inclusions.

When the cell’s internal stress level is moderate, the proteins are sequestered by the PQC to both compartments. JUNQ inclusions only develop if the quality control machinery is compromised^23^,^34^. During the rotenone treatment, the cell viability is not affected, and we therefore conclude that cellular stress levels are low compared to treatment with α-S monomers and α-S seeds. We postulate that during rotenone treatment, the PQC is still partially active and proteins in the JUNQ can be refolded or degraded to some extent. Therefore fewer JUNQ inclusions form and more non-toxic IPOD-inclusions appear.

The results presented here support the hypothesis that in neurodegenerative diseases accumulation of aggregation prone proteins in the JUNQ compartment is toxic^24^. However, although accumulation of aggregated protein in the IPOD has been suggested to prevent hazardous interactions with the cellular proteome, IPOD-inclusions may still contribute to neurotoxicity by sequestering other proteins^69^, taking up space and blocking normal transport pathways, especially within the narrow confines of the axons and dendrites (Fig. 2D).

The results obtained are not specific to the model system studied. The distribution of α-S over different PQCs observed in SH-SY5Y cells and primary neuronal cultures was also observed in tissue derived from a patient suffering from LB disease. The majority of LBs in this patient’s brain tissue was immunoreactive for α-S, but not for hsp70 and lmp2. Only a minor fraction of <5% could be assigned to the JUNQ-compartment. The large fraction of nontoxic IPOD-LBs in diseased tissue may result from the long time span between the onset of the disease and death. In time, cells containing toxic JUNQ inclusions will die. Hence, the relative number of cells containing inert and harmless IPOD inclusions is expected to increase. This suggests that when α-S is exclusively sequestered to IPOD-LBs it hardly affects cell viability.

**Figure 7:**
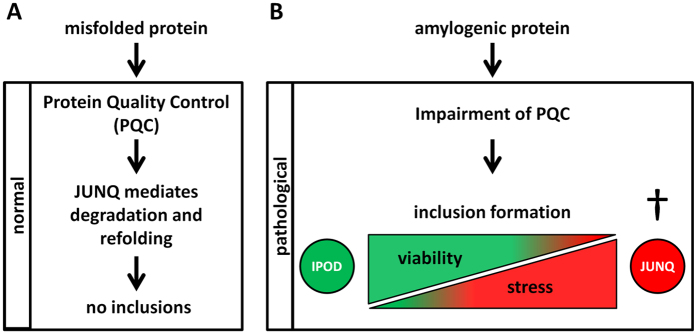
The sequestering of misfolded proteins to specific internal PQC compartments is related to internal stress level and directly affects cell viability. Misfolded cytosolic proteins can either be refolded or degraded by proteins in the JUNQ compartment or stored in the IPOD. This sorting of misfolded proteins is managed by the PQC. (**A**) When misfolded proteins appear but the cell’s internal stress level is moderate, the proteins are sequestered by the PQC to both compartments: they are either processed or temporally stored in the JUNQ where active degradation or refolding prevents the formation of proteinaceous inclusions. Alternatively, they are sequestered to the IPOD where they form insoluble inclusions. IPOD inclusions are not directly toxic, but fibrils stored in the IPOD can seed protein aggregation in other cells. (**B**) Elevated stress levels increase the amount of misfolded, cytoplasmic proteins. Hence, more misfolded proteins are sequestered to the JUNQ compartment and may saturate and exhaust the quality control machinery. The JUNQ compartment clogs, and protein degradation or refolding is impaired. Cytotoxic proteinaceous inclusions are formed in the JUNQ, this toxicity may arise from sequestration of other proteins involved in managing and processing misfolded proteins (e.g. hsp70) and accumulation of misfolded or aggregated protein in the cytoplasm^23^.

In conclusion, our findings support a model for LB formation in which misfolded α-S can be sequestered to IPOD or JUNQ inclusion sites. We speculate that cellular stress levels determine the ratio of JUNQ to IPOD-LBs, which in turn directly affects cell viability and stage or progression of disease (Fig. 7). By preventing the induction of JUNQ-inclusions and the subsequent decline of the intracellular protein quality control, cell and tissue degeneration may be averted. The presented cell model system may offer an attractive platform to develop therapeutics that target the formation, inhibition, or degradation of toxic JUNQ inclusions.

## Materials and Methods

### Recombinant protein

Expression of human wild type α-S and the 140C mutant (α-S 140C) with a single alanine to cysteine substitution at residue 140 was performed in *E. coli* B121 (DE3) using a pT7 based expression system. For α-S, the N-terminus plays a critical role in membrane binding^70^. Attaching the GFP molecule to the C-terminal end of α-S minimizes interference with both membrane binding and amyloid forming properties of α-S^71^. Details on α-S purification procedure are described elsewhere^72^. Purified protein was stored at −80 °C in aliquots until further use. α-S A140C monomers were conjugated with AlexaFluor350 maleimide following the manufacturer’s labelling protocols (Life Technologies, USA). For expression of GFP with a poly-histidine (HIS) tag, α-S with GFP-HIS tag and α-S with HIS tag, pET28A constructs were used. The constructs were expressed in *E. coli*, extracted and purified on a Ni-NTA column (Invitrogen, USA) (see Supplementary Fig. S1).

### Production of α-S fibrils and seeds

To produce short α-S fibrils that can seed aggregation, 100 μM α-S monomers were assembled into fibrils in aggregation buffer (100 mM NaCl, 50 mM Tris-HCl pH 7.4, 5 μM ThioT, 0.005% NaN_3_) at 37 °C under constant agitation (950 rpm) in a plate reader (Infinite 200 PRO multimode, Tecan Ltd., Switzerland). In the plate reader, fibril growth was followed by monitoring ThioT fluorescence (excitation 446 nm, emission 485 nm), the reaction was stopped at maximal fluorescence intensity after 5 days. The obtained α-S fibrils were dialyzed (Slide-A-Lyzer G2 Dialysis Cassettes, 10K MWCO, Pierce, USA) against 2 l of PBS containing 1% penicillin and streptomycin for 24 hours at 4 °C. Subsequently, fibril fragments were produced by sonicating the fibrils with a tip sonicator (Sonifier 250, Branson Ultrasonics Corporation, USA) on ice for 3 min. The thus obtained α-S seeds were visualized by AFM (see Supplementary Fig. S5A), aliquoted and stored at −80 °C until use. To test the ability of obtained α-S seeds to nucleate aggregation of a monomeric α-S pool, 500 nM α-S seeds were added to a solution of 25 μM α-S monomers in F-Buffer (10 mM Tris-HCl pH 7.4, 2 mM MgCl2, 1 μM CaCl2, 0.2 mM DTT, 0.5 mM ATP, 1M KCl) including 5 μM ThioT on a plate reader under constant agitation. The aggregation was stopped after 24 hours at maximum ThioT fluorescence (see Supplementary Fig. S5B). To assemble α-S-GFP or α-S 140C^alexa350^ fibrils, we used the same protocol. 100 μM protein (ratio 1:10 of α-S-GFP or α-S 140C^alexa350^ and α-S) was incubated in aggregation buffer (10 mM NaCl, 10 mM Tris-HCl pH 7.4, 0.1 mM EDTT pH 7.4) for 5 days under constant shaking (500 rpm) at 37 °C. To visualize the α-S-GFP fibrils, the protein-buffer solution was diluted 1:10 in PBS. Total internal reflection (TIRF) microscopy images were obtained by using a Nikon Ti Eclipse inverted microscope with PlanFluor 60x Ph1 DLL objective (Nikon, Japan) (see Supplementary Fig. S2).

### Atomic Force Microscopy

AFM samples were prepared by adsorbing 100 nM of α-S seeds on freshly cleaved mica for 4 min, followed by 2 washes with 100 μl of deionized water. The samples were dried under nitrogen gas passed through a 0.22 μm filter. AFM images were acquired on a Bioscope Catalyst (Bruker, Santa Barbara, CA, USA) in tapping mode using a silicon probe, NSC36 tip B with force constant of 1.75 N/m (MikroMasch, Tallin, Estonia). All images were captured with a scan rate of 0.5 Hz.

### STED microscopy

Stimulated emission depletion (STED) microscopy^73^ was employed for subdiffraction resolution fluorescence imaging on a custom-made setup. The system’s implementation is based on a supercontinuum laser source, and similar to the setup described elsewhere^74^. It is capable of acquiring one channel with confocal and two channels with STED resolution quasi-simultaneously. The supercontinuum laser source was a SC450-PP-HE system running at 1 MHz, manufactured by Fianium Ltd, UK. For beam-scanning, we used a YANUS IV scan head from Till Photonics, Germany. The objective was a Leica 100x/1.4. For imaging GFP, Alexa Fluor® 594 and Alexa Fluor® 647, we used excitation/emission wavelengths of 488 ± 3 nm/520 ± 14 nm, 586 ± 7 nm/624 ± 40 nm and 637 ± 5 nm/ 685 ± 20 nm, respectively, using optical filters from AHF, Germany. The STED wavelengths for Alexa Fluor® 594 and Alexa Fluor® 647 were set to 720 ± 10 nm, and 750 ± 10 nm, respectively. Beam powers for acquisition were 1–5 μW for the excitation beams, as measured in front of the objective. STED beam powers amounted to 1–2 mW. To reduce crosstalk, pulses for various channels were separated in time by varying optical path lengths. A home-built electronic gating device transmitted detector signals occurring at the correct time to the acquisition hardware, and rejected crosstalk signals occurring at other times. Dichroic mirrors and filters were purchased from AHF, Germany.

### Cell culture, transfection and selection of SH-SY5Y cells

SH-SY5Y cells were grown in proliferation medium, a 1:1 mixture of Ham’s F12 medium including Gibco® GlutaMAX™ and GIBCO® EBSS supplemented with 10% heat inactivated FBS and 1% Penicillin/Streptomycin. All SH-SY5Y cells used in experiments were differentiated into post-mitotic, neuron-like cells with extended dendrites and expressing neuronal marker proteins as described elsewhere^75^. In short, for differentiation, we seeded SH-SY5Y cells to 60% confluency and induced differentiation by adding starvation medium containing 1% FBS and 10 μM retinoic acid for 7 days. All chemicals were obtained from Invitrogen, USA if not indicated differently.

SH-SY5Y cell lines stably expressing α-S-GFP (α-S-GFP SH-SY5Y) were established to visualize intracellular α-S. For transfection, SH-SY5Y cells were seeded, grown until 30–50% confluency, and transfected with pEGFP-N1-α-S. DNA (250 ng/cm^2^) was diluted in Opti-MEM in reduced Serum medium (GIBCO®) including Lipofectamine® LTX Reagent with PLUS™ Reagent. The mix was incubated for 5 minutes at room temperature before adding Lipofectamine LTX. For every 250 ng of DNA, 0.5 μl of Lipofectamine LTX and 0.19 μl of Lipofectamine PLUS reagent were mixed, according to manufacturer’s protocol. After Lipofectamine LTX addition, a 30 minutes incubation at room temperature was performed. The medium of the cells was changed and the DNA, Lipofectamine LTX, and Lipofectamine® LTX Reagent with PLUS™ Reagent mix were added. After one day, the medium was changed to proliferation medium. Two days after transfection, cells were trypsinized and re-seeded in conditioned medium (1 part filtered old proliferation medium and 2 parts fresh proliferation medium). The next day, G418 (500 μg/ml) was added and cells were grown in G418 supplemented conditioned medium until selection by FACS analysis. GFP-positive cells were expanded in culture dishes and stocks were stored in liquid nitrogen. For the western blot in Figure S4, SH-SY5Y cells and GFP SH-SY5Y cells they were lysed in SDS sample buffer containing 0,1M DTT. After 5 minutes boiling and 2 minutes spinning at maximum g, samples were loaded and separated on a 12% SDS PAGE gel. The blot for α-S detection was, prior to blocking in 5% NFDM in TBS + 0.3% tween, first fixed in 0.4% PFA in PBS to improve detection. The antibody used for α-S was obtained from BD biosciences (nr610786). For the visualization of α-S, a shorter exposure and longer time of the same blot was used and compared. For GFP detection a polyclonal antibody raised in rabbit against GFP (Invitrogen A11122) was used, to visualize GAPDH the antibody sc-32233 from Santa Cruz was used.

### Primary neuron extraction and culture

The extraction and culturing of primary neuronal cells was performed as described elsewhere^76^. In short, cells were obtained from new born (P1) Wistar rat pups. Both (cortical) cerebral hemispheres were isolated in a sterile environment, minced and trypsinized. The minced hemispheres where dissociated by trituration after which the cells were ready to be plated on polyethylenimine-coated culture dishes (Acros Organics, USA) with glass bottoms or polyethylenimine coated coverslips (Sigma-Aldrich, USA) to 60% density. After 2 hours, adhered cells were washed with DMEM (Invitrogen, USA) and cultured in 900 μl serum and antibiotics-free R 12 medium^77^ at 37 °C with 5% CO_2_. All research involving animals has been conducted according to Dutch law (as stated in “Wet op de dierproeven”), and approved by DEC, the Dutch Animal Use Committee.

### α-S inclusion formation, -quantification and determination of nuclear proximity

The formation of α-S inclusions was initiated by: i) exposure to 100 μM α-S monomers, ii) exposure to 500 nM α-S seeds both for 24 hours, followed by a 4 day incubation in starvation medium and iii) exposure to 5 nM rotenone for 28 days. Cells were labelled for α-S^alexa594^ and phalloidin^alexa647^. The cell nuclei were visualized with DAPI. Labelled cells containing α-S inclusions were imaged using confocal microscopy (LSM 510, Zeiss, Germany). The α-S inclusions morphologies were classified and divided in round, oval-kidney like, amorphous or hairy. To make this classification possible the laser intensities and thresholds were adjusted in a way that for each sample the full range of possible intensities was covered and no over- or under saturated pixels were present. To be considered for shape classification the α-S inclusions had to be larger than 0.5 μm. In our shape classification we considered α-S inclusions to be 1) Oval, if the inclusion had sharp boundaries and one elliptical axis that was at least 1.5 times as long as the other. 2) Round, if the inclusions had sharp boundaries and one elliptical axis was less than 1.5 times as long as the other. 3) Hairy, if the inclusions had at least three evaginations with a length >1 μm and a width < optical resolution. 4) Amorphous, if the inclusions did not have well defined boundaries. Nuclear proximity was evaluated by ascertaining the distance of α-S inclusions to the nucleus; inclusions whose distance to the nucleus was smaller than their diameter (longest inclusion axis + shortest inclusion axis)/2) were classified as juxtanuclear.

### Immunocytochemistry, ThioS and phalloidin staining

Cell samples were washed with PBS and fixed in 3.7% paraformaldehyde/PBS solution. For immunolabelling, cells were permeabilized with 0.3% Triton X-100 and 0.1% BSA in PBS. Autofluorescence was quenched with 50 mM NH_4_Cl in PBS. Thereafter, primary antibodies (Table 1) were diluted 1:100 in goat serum dilution buffer (16% goat serum, 0.3% Triton X-100, 0.3 M NaCl in PBS) and incubated overnight. The next day, cells were washed 3 times with 0.3% Triton X-100 and 0.1% BSA in PBS at room temperature and the appropriate secondary antibodies (Table 2) were diluted 1:100 in 0.3% Triton X-100 and 0.1% BSA in PBS and incubated for 1 hour. For ThioS or phalloidin staining, fixed cells were incubated with 0.05% ThioS or 70 nM phalloidin^alexa647^ in PBS for 15 minutes. Nuclear counterstaining was performed by incubation in 300 nM 4′,6-diamidino-2-phenylindole (DAPI) in PBS for 10 minutes. After washing with PBS, samples were mounted with mounting medium (ibidi, Germany).

### Confocal Microscopy

Confocal laser scanning microscopy images were obtained using a LSM510 Confocal microscope with an 63 × oil immersion objective (NA = 1.4, Zeiss, Germany) with appropriate laser lines (Argon laser (488 nm), Helium-Neon laser (543 nm), Argon ion laser (633), Chameleon pulsed laser (340 nm, Coherent, USA). Images were taken successively. Emission was detected using appropriate dichroic mirrors and filter sets. Images were analyzed with the ZEN software 2009 (Zeiss, Germany).

### Histology

For Eosin-haematoxylin staining (HE), α-S-GFP SH-SY5Y cells were grown on cover glasses coated with collagen IV. Subsequently cells were fixed in a 0.4% PFA in PBS solution. Samples were washed and stained with eosin followed by haematoxylin, mounted with mounting medium (ibidi Germany) and imaged (E600, Nikon, Japan).

A biopsy of human mesencephalon derived from a patient diagnosed for LB disease was dehydrated in an increasing ethanol series, embedded in paraffin and cut in 5 μm sections. After rehydration, the sections were treated with formic acid and heated to 97 °C for 10 minutes in TRS buffer (Dako) followed by a treatment with 3% H_2_O_2_. Samples were immunostained for α-S^alexa555^, hsp70^alexa633^ and lmp2^alexa488^ (Tables 1 and 2). DAPI was used as counterstaining.

### Immunoblot analysis

Cells were seeded and grown to 60% confluency and differentiated in T25 culture flask (Greiner, Germany). Cells were then lysed [4× Laemmli buffer (8% SDS, 240 mM Tris-Cl, pH 6.8), 100 mM DTT] for 15 minutes at room temperature, scraped and transferred to Eppendorf tubes. Lysates were sonicated, boiled for 5 minutes and centrifuged for 2 minutes at 12000 rcf (IEC MicroMAX tabletop centrifuge). Cleared lysates were separated on a SDS-PAGE gel (12%) and blotted onto methanol activated PVDF membrane (Millipore, USA). For improved α-S detection, membranes were first fixed in a 0.4% PFA in PBS solution, then blocked in non-fat-dried-milk (5% ELK, Campina) in TBS and Tween-20 (0.3%) and incubated overnight at 4 °C with primary antibody against α-S or GAPDH. Primary antibodies were detected by goat-anti-mouse-HRP conjugated antibodies [1:8000 in TBS + tween-20 (0.3%)]. Protein Marker bands were visualized by Strep-Tactin-HRP [1:5000 in TBS + tween-20 (0.3%)] (Bio-Rad, USA). HRP-conjugated protein bands were detected by Clarity Western ECL Substrate (BioRAD, USA) and imaged on a FluorChem M imager (Protein Simple, USA).

### Viability and Metabolic activity assay

Cells were seeded in either flow channels for the viability assay (μ-slide I, ibidi Germany) or 24-well plates (Greiner Bio-One GmbH, Germany) for the metabolic activity assay, grown to 60% confluency and differentiated. For the viability assay, cells were incubated with 100 μM α-S monomers or 1 μM α-S seeds for 72 h. The viability assay was performed according to manufacturer’s instructions (Invitrogen, USA). In short, cells were washed with cold PBS and incubated with 100 μl/mL solution of propidium iodide (PI) for 15 minutes at room temperature. Subsequently, cells were washed with binding buffer. Images were obtained using an inverted fluorescence microscope (EVOS, AMG, USA).

For the metabolic activity assay, cells were incubated with 100 μM α-S monomers or 500 nM α-S seeds for 24 hours, 5 nM rotenone for 28 days, or 100 μM rotenone for 36 hours. Next, cells were treated with 0.5 mg/ml MTT (Invitrogen, USA) in medium for 4 hours at 37 °C in an atmosphere of 5% CO_2_. Before the medium was withdrawn carefully and discarded, detached cells were removed via gentle centrifugation (500 × g, 5 min) and included in the metabolic activity assay. After cell solubilization with DMSO, metabolic activity was quantified on a multiwell scanning plate reader by measuring the absorbance at 540 nm with background subtraction at 690 nm (Tecan Ltd, Switzerland).

## Additional Information

**How to cite this article**: Raiss, C. C. *et al.* Functionally different α-synuclein inclusions yield insight into Parkinson’s disease pathology. *Sci. Rep.* **6**, 23116; doi: 10.1038/srep23116 (2016).

## Supplementary Material

Supplementary Information

## Figures and Tables

**Table 1:** Primary antibodies.

Antibody	Epitope	Host species	Source
α-S	15–123	mouse	BD biosciences
α-S	123–140	guinea pig	Abcam
α-S	96–140	rabbit	Santa Cruz Biotechnology
α-S pSer129	Ser 129	rabbit	Santa Cruz Biotechnology
20S LMP2	lmp2	rabbit	Stressgen
hsp70	hsp70	mouse	Abcam
GFP	GFP	rabbit	Invitrogen
GAPDH	GAPDH	mouse	Invitrogen

**Table 2:** Secondary antibodies.

Host	Anti	Conjugate	Source
goat	mouse	HRP	Sigma
goat	mouse	Alexa488	Invitrogen
goat	mouse	Alexa555	Invitrogen
goat	mouse	Alexa594	Invitrogen
goat	mouse	Alexa633	Invitrogen
goat	rabbit	Alexa488	Invitrogen
goat	rabbit	Alexa555	Invitrogen
goat	rabbit	Alexa633	Invitrogen
goat	rabbit	HRP	Invitrogen
goat	guinea pig	Alexa555	Invitrogen
