# Supplementary material for: Functionally different α-synuclein inclusions yield insight into Parkinson’s disease pathology
Source: Sci Rep. 2016 Mar 17;6:23116. doi: 10.1038/srep23116 (PMC4794800; doi:10.1038/srep23116)
Supplement: Supplementary Information [file srep23116-s1.pdf]

Functionally different α-synuclein inclusions yield insight into Parkinson's disease pathology

Christian C. Raiss[1], Theresa S. Braun[1], Irene B. M. Konings[1], Heinrich Grabmayr[4], Gerco C. Hassink[2], Arshdeep Sidhu[1], Joost le Feber[2], Andreas R. Bausch[4], Casper Jansen[3], Vinod Subramaniam[1,5], and Mireille M. A. E. Claessens[1]

Supplementary Information (SI)

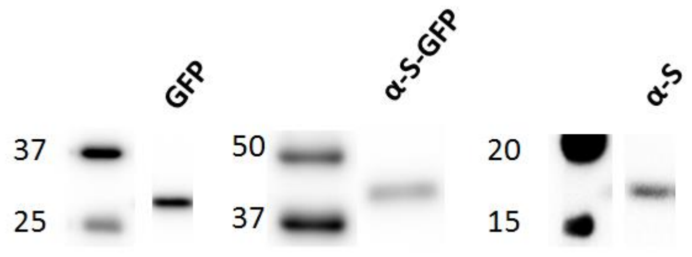

Supplementary Fig. S1. Immunoblot of recombinantly expressed GFP, α-S-GF, and α-S. The molecular weight of recombinantly expressed α-S-GFP and α-S is comparable to protein expressed in SH-SY5Y cell lysate (see Supplementary Fig S3 A).

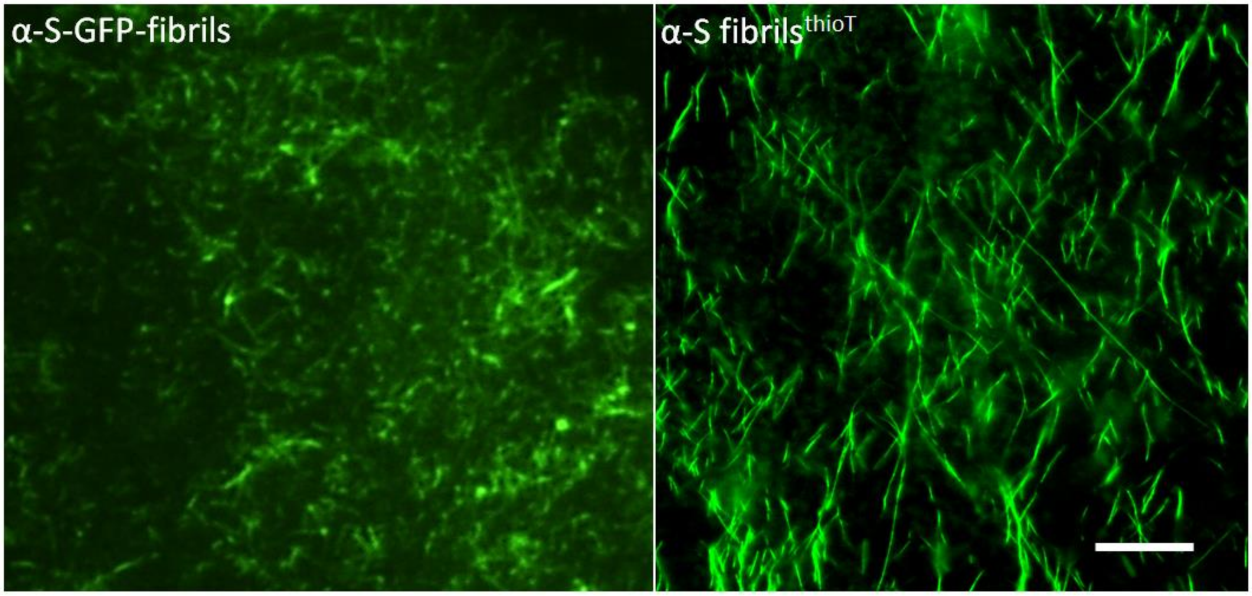

Supplementary Fig. S2. α-S-GFP assembles *in vitro* into fibrils. Purified recombinantly expressed α-S-GFP was able to aggregate into amyloid fibrils when mixed in a 1:10 ratio with untagged WT-α-S (left image). Compared to pure WT-α-S fibrils (stained with ThioT, right image) the WT-α-S fibrils seemed relatively short; TIRF microscopy; scale bar 10 µm.

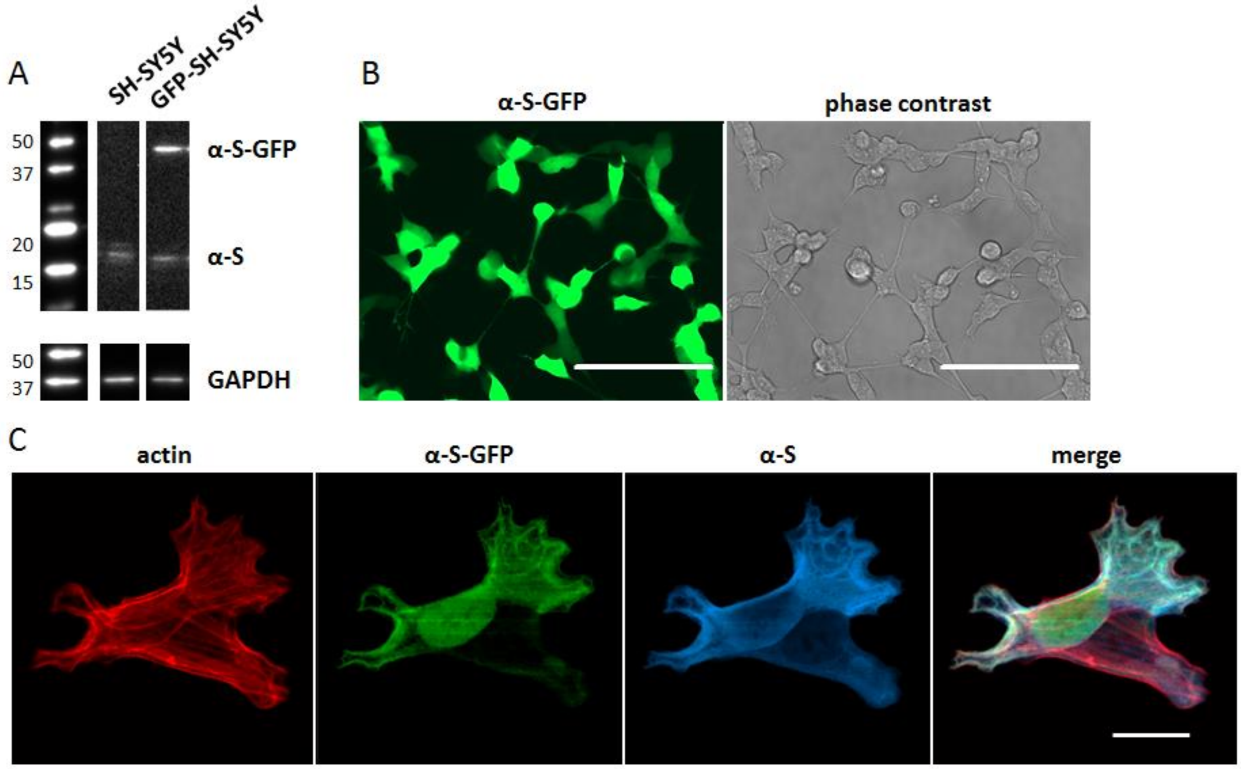

Supplementary Fig. S3. SH-SY5Y cells stably express (exogenous) α-S-GFP. A) Immunoblot of α-S in cell lysate from SH-SY5Y and αS-GFP SH-SY5Y cells. αS-GFP SH-SY5Y lysate shows immunoreactivity for α-S and α-S-GFP. The expression levels of untagged α-S are comparable to the levels observed in untransfected SH-SY5Y cells. B) GFP fluorescence from αS-GFP SH-SY5Y cells. Cells contain α-S-GFP in different concentrations, judging by different fluorescent intensities. To visualize the low α-S-GFP levels of some cells, GFP intensity from cells with high expression levels was saturated; scale bar: 100 µm. C) Cells with different endogenous α-S expression level.α-S-GFP fluorescence shows colocalization to α-S antibody fluorescence; confocal image of αS-GFP SH-SY5Y cells with fluorescent labelling for actinalexa647 and α-Salexa594; scale bar: 10 µm.

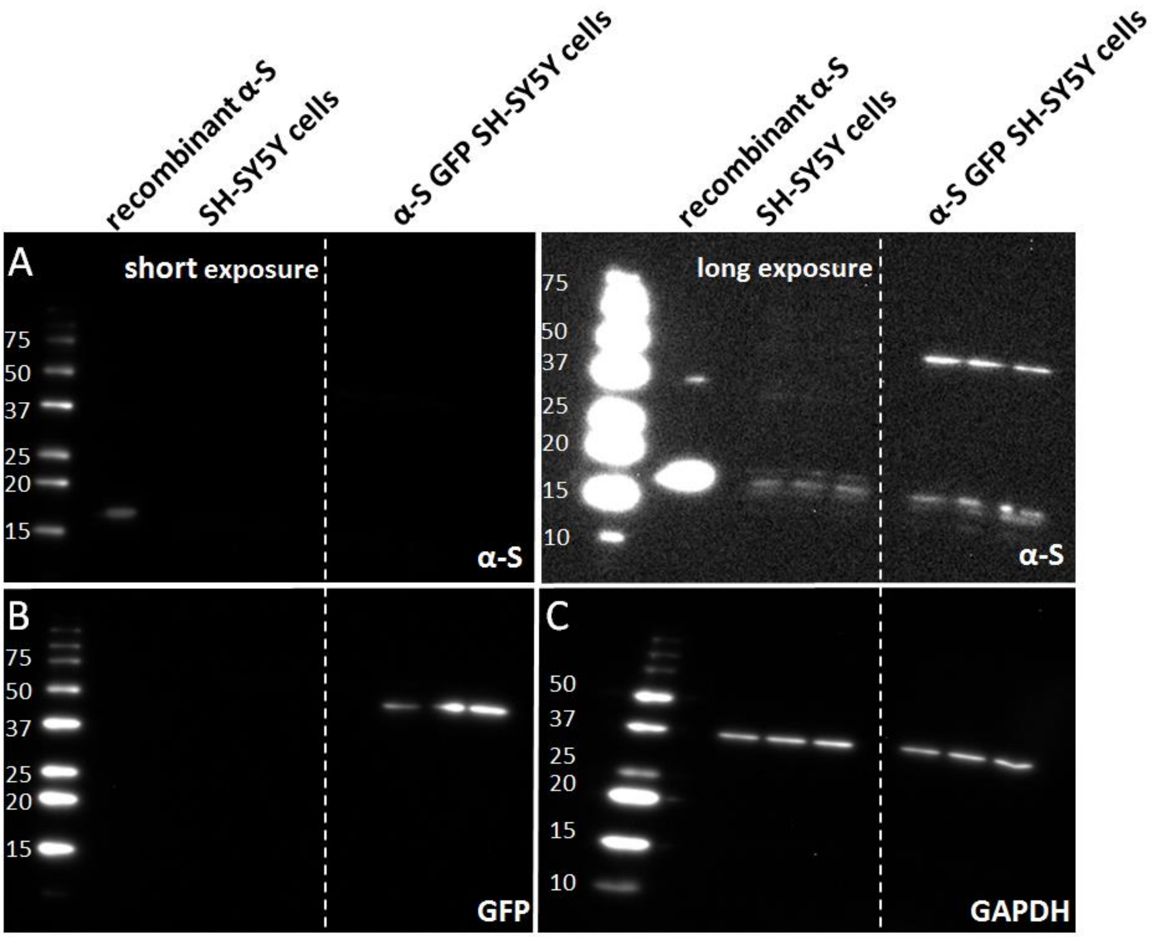

Supplementary Fig. S4. Cell lysates of wild type and α-S-GFP SH-SY5Y cells show no cleavage of GFP from α-S-GFP. A) α-S is clearly visible in lysates of both cell types on western blot after long exposure during imaging. In the lysate of α-S-GFP SH-SY5Y cells, α-S and α-S-GFP bands can be detected. B) Antibodies against GFP show that GFP is not cleaved form α-S-GFP. C) Control blot for GPADH.

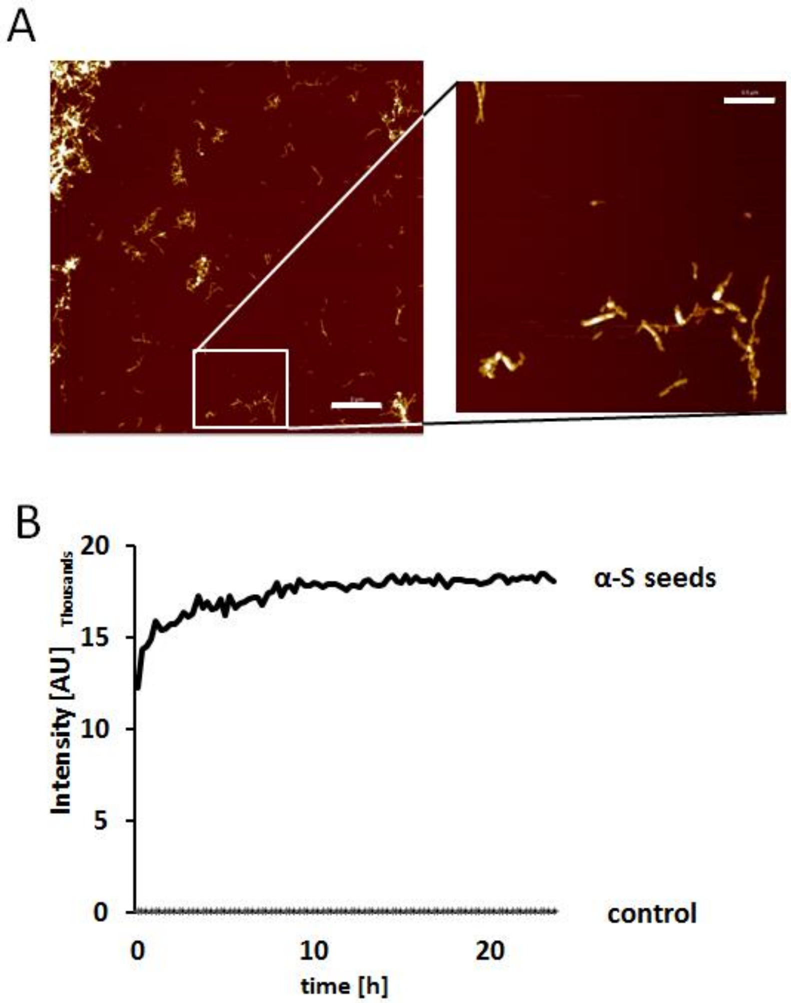

Supplementary Fig. S5. Visualization of α-S seeds and effect of seeds on α-S fibrillization. Sonicated α-S fibrils were used to initiate aggregation of an α-S monomer pool. A) After fragmentation of long α-S fibrils, α-S aggregates were imaged by AFM. AFM height images of ThioT labelled α-S seeds reveal small µm-sized α-S fibril pieces; scale bar: 2 µM and 0,5 µm. B) Kinetics of α-S aggregation in the presence of 500 nM seeds measured by ThioT fluorescence reveal direct aggregation of a 25 µM solution of α-S monomers after addition of α-S seeds. No aggregation was observed for the control without α-S seeds.

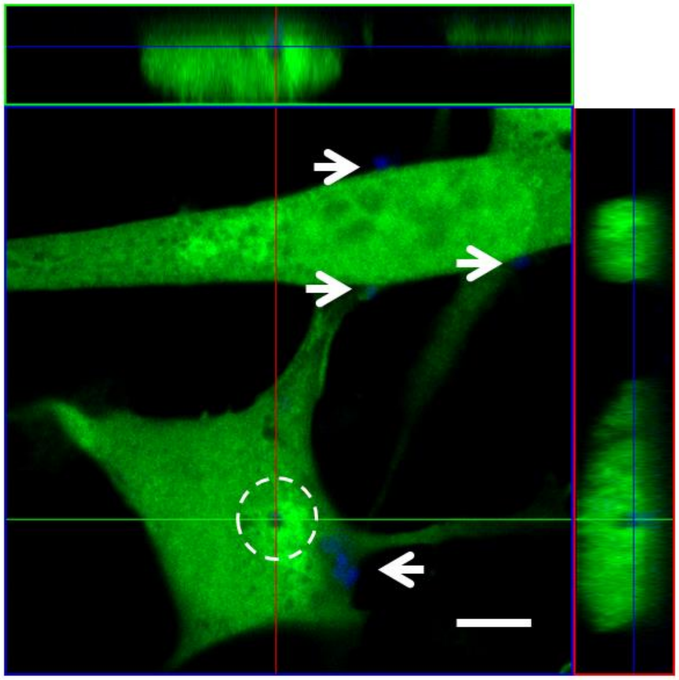

Supplementary Fig. S6. Fragmented α-S fibrils are internalized by cells upon addition to medium. After differentiation, GFP-SH-SY5Y cells were exposed to fluorescently labelled α-S-fibril fragments^{alexa350}. Shortly after addition, cellular uptake was observed; confocal microscopy, orthographic image, incorporated α-S fibrils are indicated by the dashed white circle. Fibril pieces sticking to the outer membrane are indicated with white arrows, scale bar: 5 µm.

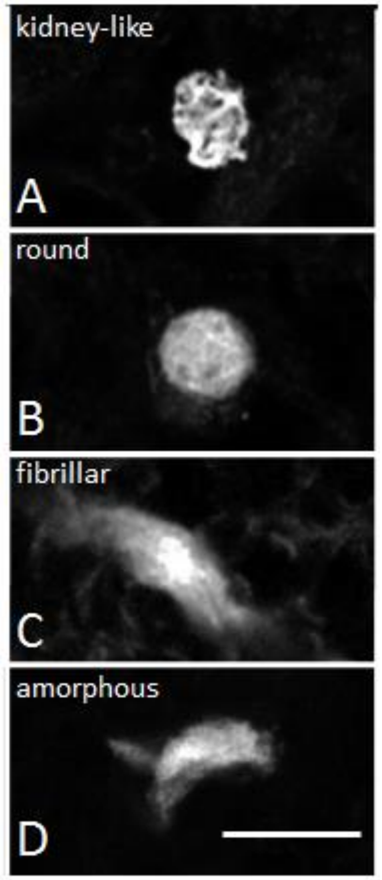

Supplementary Fig. S7. α-S inclusion morphologies found *in vitro* were also observed *in vivo*. Similar α-S inclusion morphologies induced in SH-SY5Y cells are found in a diseased human brain sample. A biopsy of a sample of mesencephalon of a patient suffering from clinical dementia was immunolabelled for α-S^{A594}. (A) Kidney-like, (B) round, (C) with fibrillar α-S fibrils and (D) amorphous LBs were observed, STED microscopy; scale bar 3 µM.

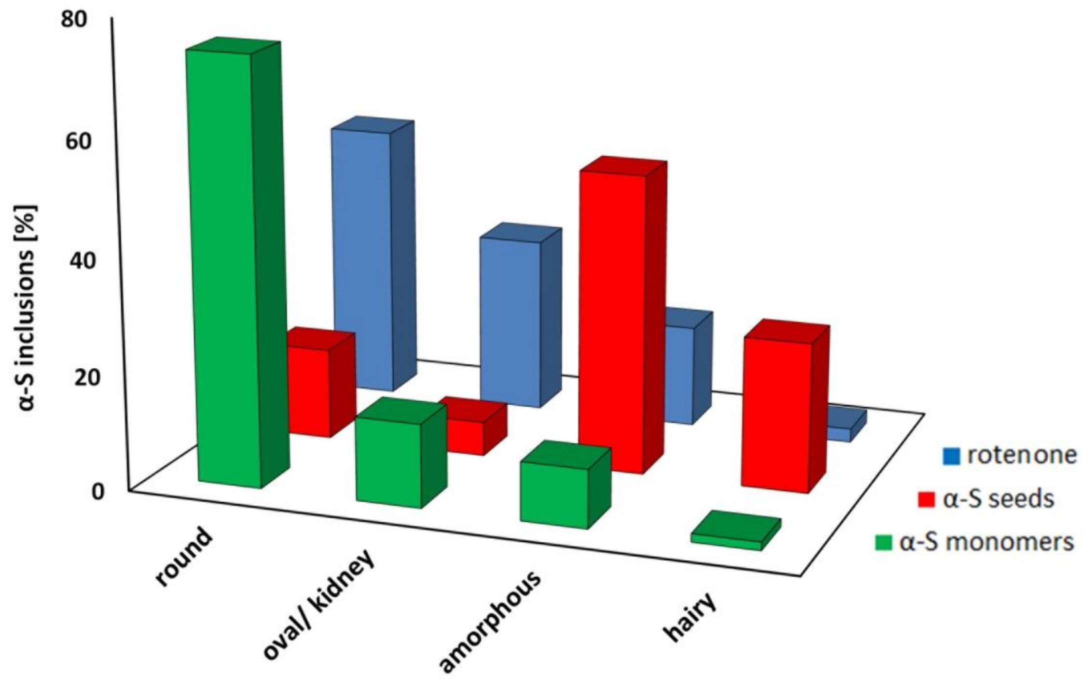

Supplementary Fig. S8. α-S inclusion morphology in primary neurons depends on induction method. After induction, α-S inclusions are divided into subgroups, according to their morphology. α-S inclusions are induced in primary neurons and cells treated with rotenone (N=84), α-S monomers (N=69), α-S seeds (N=50) are grouped according to morphology (round, oval, amorphous, hairy). α-S inclusions induced by α-S monomers are mainly round (74 %) and dense. After with treatment with seeds α-S inclusions were mainly amorphous (52 %). Induction with rotenone prevalently leads to round and oval inclusions (49 and 31 %).

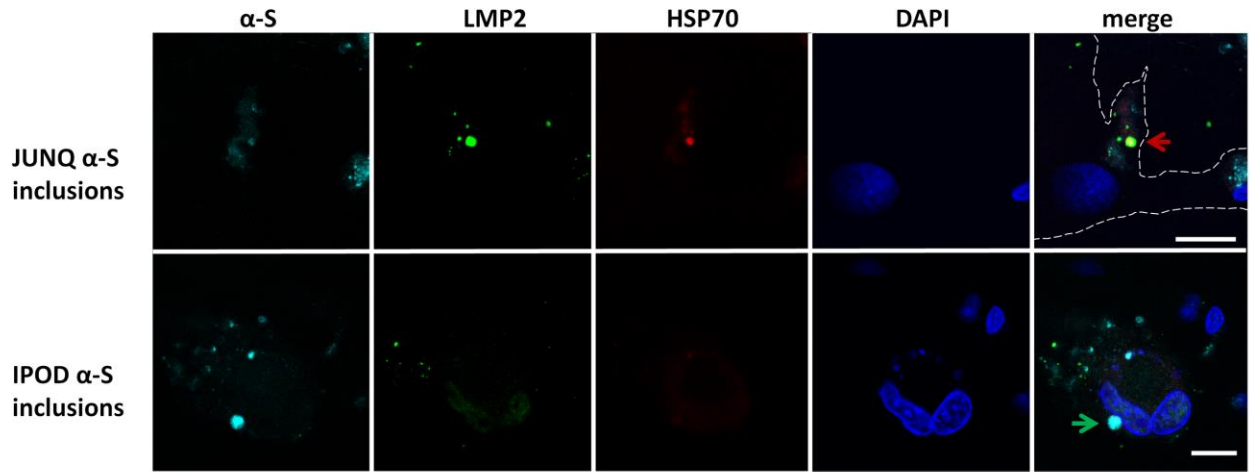

Supplementary Fig. S9. α-S sequesters in IPOD and JUNQ-inclusions in rat primary neuronal cells. α-S inclusions are induced in rat primary neurons after exposure to 100 µM of α-S monomers for 24 hours. Immunostaining was performed for α-$S^{alexa555}$ (cyan), $lmp2^{alexa488}$ (green), $hsp70^{alexa633}$ (red) and counterstaining DAPI (blue). Two different α-S inclusion types can be observed judging by protein colocalization. hsp70 and lmp2 colocalize with α-S in JUNQ-inclusionss (top row, red arrow), in other α-S inclusions, no colocalization can be observed and are therefore categorized as IPOD-inclusions (bottom row, green arrow); dashed white line defines cell shapes; scale bar: 10 µm.

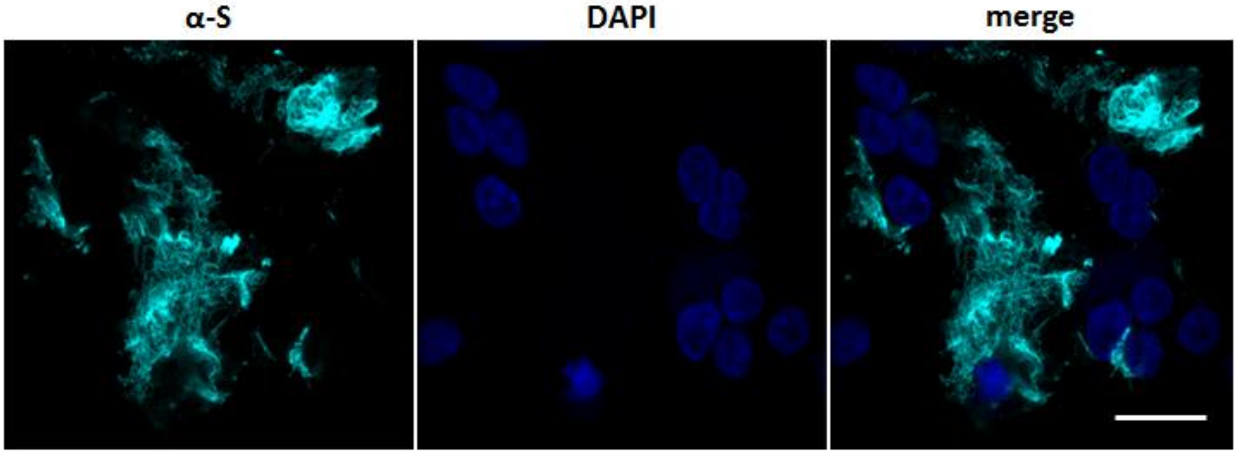

Supplementary Fig. S10. α-S monomers aggregate to α-S fibrils in medium containing cells. 24 hours after addition of 100 µM α-S monomers to SH-SY5Y cells, immunolabelling for α-S^{alexa555} (cyan) and counterstaining DAPI (blue) the formation of α-S fibrils in the medium that stick to the cells was visible; confocal microscopy, scale bar: 20 µm.
